# Molecular Simulation on the Thermal Stability of Meta-Aramid Insulation Paper Fiber at Transformer Operating Temperature

**DOI:** 10.3390/polym10121348

**Published:** 2018-12-05

**Authors:** Chao Tang, Xu Li, Zhiwei Li, Wenxin Tian, Qu Zhou

**Affiliations:** 1 College of Engineering and Technology, Southwest University, Chongqing 400715, China; swugzlx@163.com (X.L.); ccqepc@163.com (Z.L.); 13657601997@163.com (W.T.); zhouqu@swu.edu.cn (Q.Z.); 2 Zunyi Power Supply Bureau, Zunyi 563000, China; 3 Wuxi County Power Supply Branch of State Grid Chongqing Electric Power Company, Chongqing 405800, China

**Keywords:** meta-aramid insulation, thermal stability, mechanical parameter, chain motion, hydrogen bonding

## Abstract

The influence of the thermal field of a transformer during operation on the thermal stability of meta-aramid insulation paper was studied through molecular dynamics simulations. Models of the crystalline and amorphous regions of meta-aramid fibers were constructed using known parameters. The model of the crystalline area was verified by comparing X-ray diffraction results with experimental data. The reasonableness of the simulation results was judged by the variation of energy, temperature, density, and cell size in relation to the dynamic time. The molecular dynamics simulations revealed that the modulus values in the crystalline regions were two to three times higher than those in the amorphous regions at various temperatures. In addition, the incompressibility, rigidity, deformation resistance, plasticity, and toughness of the crystalline regions were obviously higher than those of amorphous regions, whereas the toughness of the amorphous regions was better than that of the crystalline regions. The mechanical parameters of both the crystalline and amorphous regions of meta-aramid fibers were affected by temperature, although the amorphous regions were more sensitive to temperature than the crystalline regions. The molecular chain motion in the crystalline regions of meta-aramid fibers increased slightly with temperature, whereas that of the amorphous regions was more sensitive to temperature. Analyzing hydrogen bonding revealed that long-term operation at high temperature may destroy the structure of the crystalline regions of meta-aramid fibers, degrading the performance of meta-aramid insulation paper. Therefore, increasing the crystallinity and lowering the transformer operating temperature may improve the thermal stability of meta-aramid insulation paper. However, it should be noted that increasing the crystallinity of insulation paper may lower its toughness. These study results lay a good foundation for further exploration of the ways to improve the performance of meta-aramid insulation paper.

## 1. Introduction

Aramid paper was developed by Du Pont in the 1960s and called Nomex paper. Aramid paper was produced from Nomex short-cut fibers and aramid pulp as raw materials using an oblique net papermaking wet method [1]. Although aramid fibers are synthesized artificially, their structure and that of naturally occurring cellulose both include regions with crystalline structure and amorphous structure.

The crystalline content of meta-aramid fibers is low; they possess an amorphous content of 75–80% [2]. The crystallinity of aramid short-cut fibers and pulp were measured by Liao Ruijing [3]. The results showed that the crystallinity of aramid short-cut fibers was 13.17%, whereas the crystallinity of aramid pulp was only 1.53%. In contrast, the crystallinity of self-made aramid insulation paper is much higher [1]; after hot pressing, the crystallinity of self-made aramid paper is 35.04%, while that of Nomex paper is 43.43%, and the crystallinity of self-made pure aramid pulp paper after hot pressing is 7.2%. The crystallinity of meta-aramid fibers prepared by Zhiqing [4] ranged from 29% to 41%. Therefore, the amorphous regions represent the major component of the molecular structure of both Nomex paper, which has the best performance in the industry, and aramid insulation paper prepared in a laboratory. Both the crystalline and amorphous regions in insulation paper are important to its performance. However, the performance of the crystalline and amorphous regions of meta-aramid fibers has not been comprehensively studied, particularly their microscopic behavior at the molecular and atomic levels. 

Aging and the performance improvement of polymers have become hot research topics [5,6,7]. Thermal aging is one of the major types of aging of insulation paper inside a transformer. In recent years, numerous studies have focused on the thermal aging mechanism of insulation paper. Although the aging process and its mechanism on oil–paper in transformers have been investigated [8,9,10,11,12,13,14,15,16,17,18], the thermal stability of meta-aramid insulation paper in transformers at operating temperature has not been analyzed. Therefore, it remains important to examine the thermal stability and thermal aging mechanism of crystalline and amorphous regions in meta-aramid insulation paper. 

In this paper, a molecular dynamics simulation method [19,20,21,22,23,24,25,26,27] is used to conduct a comparative analysis of the influence of a thermal field on the thermal stability of crystalline and amorphous regions of meta-aramid fiber from the perspective of an actual thermal field environment inside a transformer. First, we construct a model of meta-aramid with crystalline and amorphous regions using reported experimental data. Then, we use a molecular dynamics method to conduct geometry optimization, annealing of the model, and dynamics simulations. Finally, we analyze the influence of different temperatures on the mechanical parameters, chain motion, and hydrogen bonding of the model. This paper lays a foundation for further study on how to improve the performance of meta-aramid insulation paper.

## 2. Materials and Methods

### 2.1. Model Construction

#### 2.1.1. Construction of a Model of the Crystalline Area of Meta-Aramid Insulation Paper

**Figure 1:**
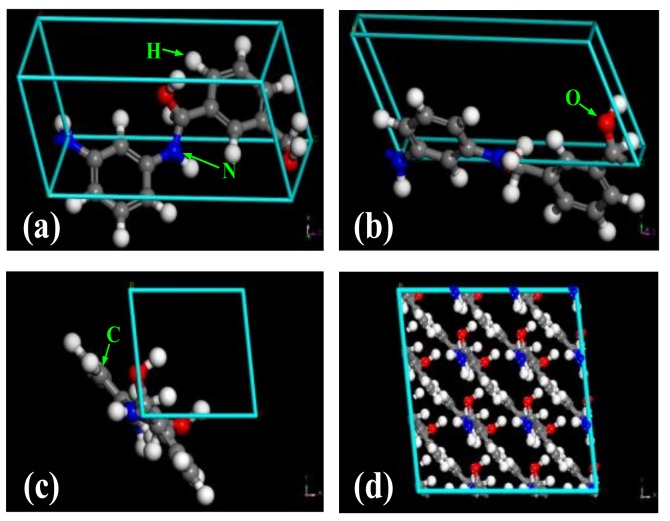
Meta-aramid fiber crystal model. (**a**) and (**b**) Side views of the unit cell in the meta-aramid fiber crystalline area, (**c**) front view of the unit cell in the meta-aramid fiber crystalline area, and (**d**) front view of the supercell.

In molecular dynamics, the first and one of the most major steps is model construction; the reliability and effectiveness of simulation results depend on model construction and processing. It is known from X-ray diffraction (XRD) analysis that the crystal structure of meta-aramid fibers is triclinic with specific unit cell parameters of *a* = 0.527 nm, *b* = 0.525 nm, *c* = 1.13 nm (fiber axis), *α* = 111.5°, *β* = 111.4°, and *γ* = 88.0° [28,29]. In addition, the microstructure of meta-aramid fibers is fibrillar. The chain number of meta-aramid fibers in the unit cell is taken to be one, and it was calculated from the crystal structure that the density of the crystalline area is *ρ* = 1.47 g/cm^3^. When meta-aramid fibers are crystallized, each molecular unit shrinks by 0.1 nm, and the angle between the planes of the benzene ring and adjacent amino group is about 30°. In addition, the space group of meta-aramid fibers is P1 [29]. The model that we constructed based on this reported information is shown in Figure 1a–c. Fractional atomic coordinates were used in the model of the crystalline area of meta-aramid fibers constructed in this paper. All models are created using the Materials Studio 5.0 Building Tool software.

Tanala et al. [30] calculated a crystal model of cellulose Iβ based on reported data [31]. They found that the dimensions of the force field and model influenced the calculated result for a tensile modulus by calculating the mechanical performance of models with different sizes: 1 × 1 × 10 and 4 × 4 × 10 unit cells. It was pointed out in their paper that a supercell is more rational than a primitive cell. Therefore, this paper uses a supercell model to calculate the crystal parameters of meta-aramid fibers. The 3 × 3 × 2 supercell model constructed using a supercell module is shown in Figure 1d. The supercell has unit cell parameters of *a* = 1.581 nm, *b* = 1.575 nm, *c* = 2.26 nm, *α* = 111.5°, *β* = 111.4°, and *γ* = 88.0°. In the constructed crystal models, the chain numbers in the unit cell and supercell are one and nine, respectively.

#### 2.1.2. Assessment of the Constructed Model

**Figure 2:**
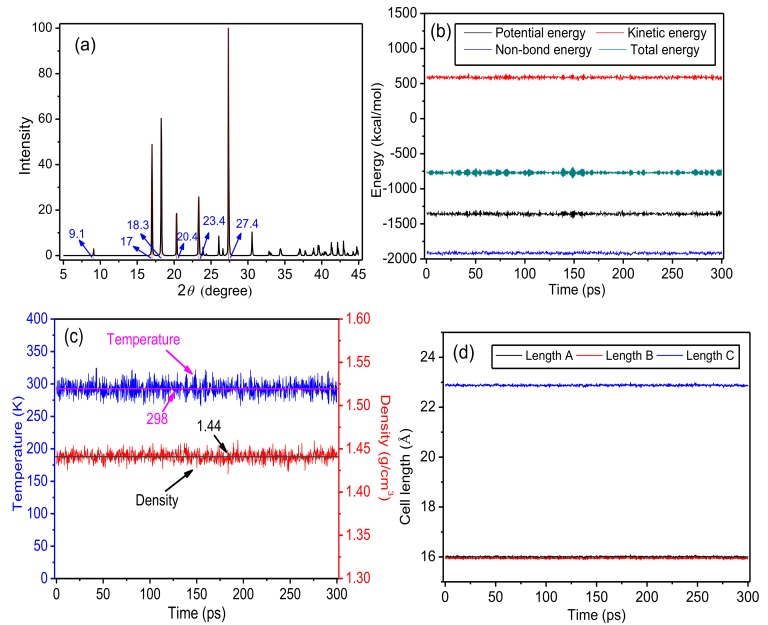
Parameters of the meta-aramid fiber crystal model. (**a**) X-ray diffraction (XRD) pattern of the supercell, (**b**) change of energy during the simulation (1 kcal/mol = 6.9477 × 10^−21^ J), (**c**) changes of temperature and density during the simulation, and (**d**) change of the unit cell length during the simulation (1 Å = 10^−1^ nm).

To verify the model, the powder diffraction module in a Reflex diffractometer was used to conduct XRD experiments. XRD parameters were set as follows: the 2*θ* range was 5°–45°, the step size was 0.05°, and copper was used as the X-ray source. The XRD pattern for the supercell, which is shown in Figure 2a, contained peaks at 9.1°, 17°, 18.3°, 20.4°, 23.4°, and 27.4°. The reported [32] XRD pattern for meta-aramid fibers contained diffraction peaks at 11°, 18°, 24°, and 28°. The difference of crystallinity of different fiber samples was detected by comparing the intensities of corresponding diffraction peaks, which revealed that the results in this paper are similar to Wen Yang [32]. Therefore, the model of the crystalline area of meta-aramid fibers that is constructed in this paper is reasonable.

The stability of a model structure needs to be assessed after construction. In this paper, the stability of the model was determined by examining the changes of energy, temperature, density, cell length, and other parameters of the model during the molecular dynamics simulation time. In the molecular dynamics simulations, energy and temperature fluctuated by 5–10%, showing that the model structure was successfully optimized, and the system reached a balanced state [33,34], allowing subsequent dynamics simulations to be conducted. The changes of crystalline area parameters of the model after geometry optimization and annealing during the simulation time at room temperature (298 K, 25 °C) are shown in Figure 2b–d.

Figure 2b–d respectively reveal that the energy, temperature, and density of the model all slightly fluctuated during the simulation time. The cell length of the model also fluctuated. Figure 2c illustrates that the steady-state values of temperature and density are 298 K and 1.44 g/cm^3^, respectively. The simulated density is slightly lower than the calculated value by Wen Yang [32], which resulted from the flaccid supercell structure during the relaxation process. The steady-state cell lengths along the X, Y, and Z axes are 1.598 nm, 1.594 nm, and 2.285 nm, respectively. These values are slightly larger than those of the initial model, which again results from the flaccid model structure during the relaxation process. Generally, the changes of the unit cell parameters of the model during the simulation time are small. After geometry optimization and annealing, the energy, temperature, density, and cell length of the model of the crystalline area of meta-aramid fibers fluctuated slightly during the simulation time. Therefore, it is believed that the model reached a balanced state, allowing further simulations to be conducted.

#### 2.1.3. Construction of a Model of the Amorphous Area of Meta-Aramid Insulation Paper

**Figure 3:**
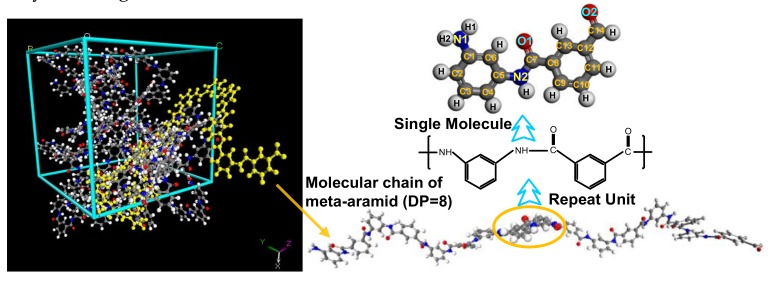
Model of the amorphous area of meta-aramid fibers.

The degree of polymerization (DP) of long polymer chains is high; for example, the average DP of cellulose in the insulation paper of new transformers is about 1000 [35]. The molecular weight of meta-aramid fibers is 60,000–90,000 [36], and the molecular chains have a high DP. However, in computer simulations, it is unnecessary to use very long chains during the computing process, which saves computing time and aids the analysis of the main mechanisms governing a system. For example, Mazeau et al. [37] used cellulose chains with different DPs to construct an amorphous model. Their simulation results showed that the models constructed with different chain lengths displayed similar molecular conformations and physicochemical properties. The visualizer module has been used to construct a meta-aramid fiber model with a DP of 10 for the selection and survey of PCFF (polymer consistent force field) and COMPASS (Condensed-phase Optimized Molecular Potentials for Atomistic Simulation Studies) force fields [33]. Another study constructed a meta-aramid fiber model with a DP of four to investigate the interfacial interactions between aramid and silicon dioxide [38]. Previous results show that it is unnecessary to construct a molecular model with the same DP as that of reality, as long as it is guaranteed that the constructed model and selection of simulation parameters are reasonable. For the model of the amorphous area of meta-aramid fibers constructed in this work, the DP was eight, the chain number was eight, and the initial density was 1.0 g/cm^3^. An amorphous cell module was used to construct the model. The constructed model is depicted in Figure 3. The changes of the energy, temperature, density, cell length, and other parameters during the dynamics simulation time were similar to those of the model of the crystalline area, so they are not given here.

### 2.2. Simulation Details

Materials Studio (Accelrys, San Diego, CA, USA) software was used to conduct the simulations. The Build Crystals module was employed for model construction, and the Forcite module was used for geometry optimization, annealing, and dynamics simulations with periodic boundary conditions. After each model was constructed, its energy was high, and its structure was in an unstable state. Thus, it was necessary to conduct geometry optimization and then annealing for the various models. The purpose of annealing was to make the model structures more realistic. Fine was selected as the quality of geometry optimization, and the Smart algorithm was used during the optimization process with a maximum iterative step number of 5000, annealing period of 10, and initial temperature of 300 K. The mid-cycle temperature of each period is set as 900 K, and the number of heating ramps per cycle was 10. After optimization and annealing, the rationality of the model structure needed to be assessed. Molecular dynamics simulations were conducted if the model was rational; otherwise, the model was optimized and annealed again until it became rational. Dynamics simulations were conducted for 300 ps in the NVT (Number of particles, Volume and Temperature) ensemble with a step length of 1 fs, truncation radius of 9.5 Å, and spline width of 1 Å. The NVT ensemble was then changed to the NPT (constant pressure and temperature) ensemble using the temperature control method of Nose and pressure control method of Berendsen. The pressure was set as standard atmospheric pressure, the simulation time was 300 ps, and dynamics information was collected every 500 steps. The ambient temperature inside a transformer is generally 363 K, and may increase to about 423 K for short periods. Therefore, the investigated temperature range was 343–423 K. During the simulation process, the target temperatures were 343–423 K with an increment of 20 K. Therefore, five systems were simulated for each model.

## 3. Mechanical Parameters

### 3.1. Mechanical Parameters of the Crystalline Regions of Meta-Aramid Insulation Paper

The mechanical parameters of polymers are very important parameters [39]. The change of the mechanical parameters of insulation paper over time is a result of the long-term action of both thermal and electric fields inside the transformer. Therefore, this change can reflect the change of thermal stability of the insulation paper to a certain degree. The general relationship between stress and strain for solid materials can be expressed by the generalized Hooke law considering elasticity [40]. Various mechanical parameters of materials can be calculated based on elasticity, including bulk modulus (*K*), shear modulus (*G*), tensile modulus (*E*), *K/G* value, Poisson ratio (*ν*), and Cauchy pressure (C_12_–C_44_). These parameters can be used to show the different mechanical properties of a material. The relevance and calculation method of these parameters have been reported elsewhere [17]. The mechanical parameters of crystalline regions of meta-aramid fibers at different temperatures are summarized in Table 1. Data from the last 20 frames in the trail file were used for statistical analyses.

Except for C_35_, the values of C_15_, C_25_, and C_46_ calculated for various temperature conditions were close to 0, showing that the meta-aramid fiber crystals are not strongly anisotropic elastomers. Three groups were observed: C_11_, C_22_, and C_33_ in Group 1; C_44_, C_55_, and C_66_ in Group 2; and C_12_, C_13_, and C_23_ in Group 3. The difference between the values in groups 2 and 3 was small, whereas the variation between the values in Group 1 was large, showing that the crystalline regions of meta-aramid fibers behave as anisotropic elastomers. The Cauchy pressure is positive, showing that the toughness of crystalline regions of meta-aramid fibers is high. 

The mechanical parameters of crystalline regions of meta-aramid fibers are similar at different temperatures, showing that the influence of temperature on the mechanical performance of crystalline regions of meta-aramid fibers is small within the operating temperature range of a transformer. However, the bulk modulus, shear modulus, and tensile modulus at 343 K are higher than those at other temperatures, showing that the incompressibility, rigidity, and capacity to resist deformation of the crystalline regions of meta-aramid fibers are weakened at this temperature. Of all the investigated mechanical parameters of the crystalline regions of meta-aramid fibers, the change of *ν* is the smallest; that is, the plasticity of these regions is only slightly influenced by temperature.

### 3.2. Mechanical Parameters of the Amorphous Regions of Meta-Aramid Fibers

The mechanical parameters of amorphous regions of meta-aramid fibers at different temperatures are given in Table 2. The C_15_, C_25_, C_35_, and C_46_ values obtained at various temperatures are close to 0, showing that the amorphous regions of meta-aramid fibers are not strongly anisotropic elastomers. The values for the groups of C_11_, C_22_, and C_33_; C_44_, C_55_, and C_66_; and C_12_, C_13_, and C_23_ are very close, indicating that the amorphous regions of meta-aramid fibers are isotropic elastomers. This is different from the crystalline regions of the meta-aramid fibers, which are anisotropic elastomers; this difference is caused by their different molecular arrangements.

Various modulus values in Table 2 decreased with increasing temperature, showing that the rise of temperature weakens the incompressibility, rigidity, and the capacity to resist the deformation of the amorphous regions. The change of Poisson ratio is generally small, showing that the plasticity of the amorphous regions is only slightly influenced by temperature. The values of Cauchy pressure at different temperatures are positive and increase with temperature, revealing that the elevated temperature increases the fragility of amorphous regions of meta-aramid fibers and strengthens their toughness. The change of *K*/*G* value with temperature is not obvious, showing that the toughness of the amorphous regions is not greatly influenced by temperature.

The modulus values of crystalline regions are two to three times higher than those of the amorphous regions at the various temperatures. This shows that an increased degree of crystallinity can improve the mechanical performance of meta-aramid insulation paper in many ways. In particular, the incompressibility, rigidity, and the capacity of the crystalline regions to resist deformation are obviously higher than those of the amorphous regions. The Poisson ratios and *K*/*G* values of the crystalline regions are obviously higher than those of the amorphous regions, revealing that the plasticity and toughness of the crystalline regions are obviously better than those of the amorphous regions. Meanwhile, the Cauchy pressure values of the amorphous regions are obviously higher than those of the crystalline regions, showing that the toughness of the crystalline regions is better than that of the amorphous regions. Thus, the crystalline and amorphous regions of meta-aramid fibers have different performances under a thermal field. Within the temperature range that was used in this paper, the crystalline regions maintain pretty good mechanical performance at the different temperatures, and their modulus values are not greatly influenced by temperature. In comparison, the amorphous regions are more sensitive to temperature; the higher the temperature, the lower the modulus value. This is because the crystalline regions have a pretty stable molecular structure, whereas the molecular chains of the amorphous regions have a low degree of regularity or even random arrangement.

## 4. Chain Motion

### 4.1. Chain Motion of Crystalline Regions of Meta-Aramid Fibers

Chain motion in polymers strongly influences their thermal stability. Mean square displacement (MSD) can be used to characterize the chain motion of a polymer; the larger the slope of an MSD time curve, the more severe the chain motion of a polymer, and the poorer its thermal stability. Curves with the same trend can be directly judged based on values; namely, the larger the MSD value, the greater the chain motion. The relation between MSD and time [41,42] can be shown as:

(1) $$MSD=\langle |{\vec{r}}_{i}(t)-{\vec{r}}_{i}(0){|}^{2}\rangle$$

In Equation (1), ${\vec{r}}_{i}(t)$ is the position vector of atom *i* at time *t* in the system, and ${\vec{r}}_{i}(0)$ is the initial position vector of the atom.

**Figure 4:**
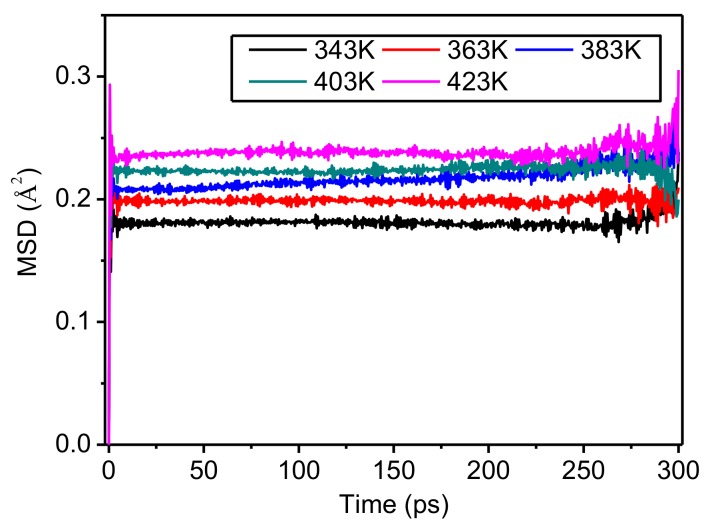
Mean square displacement (MSD) of crystalline regions of meta-aramid fibers at different temperatures.

Figure 4 presents MSD time curves for crystalline regions of meta-aramid fibers at different temperatures. The trend of the chain motion of molecules in the crystalline regions over time is the same at different temperatures. With rising temperature, the chain motion of crystalline regions of meta-aramid fibers increased slightly. While the increase of chain motion is small, it shows pretty strong temperature dependence, which is especially obvious at about 250 ps. The main reason for this is that the influence of temperature on the physicochemical properties of crystalline regions of meta-aramid fibers was small in the temperature range studied here, showing that the crystal structure of meta-aramid fibers has good stability when exposed to the operating temperature inside a transformer for a long period.

**Figure 5:**
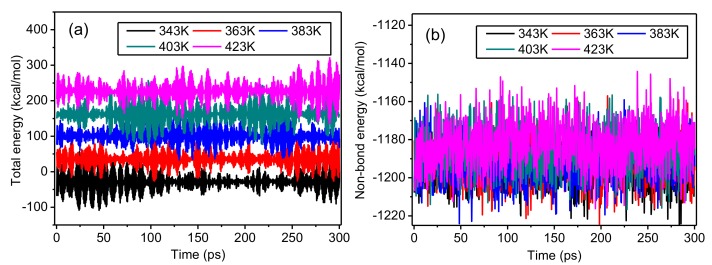
Changes of (**a**) total energy and (**b**) non-bond energy during the simulations at different temperatures.

The changes of total energy and non-bond energy during the simulations at different temperatures are displayed in Figure 5. Figure 5a reveals that with lengthening simulation time, the model energy remained in dynamic balance, showing that the previous optimization achieved its expected purpose. The molecular kinetic energy increases with temperature, and the total energy of the model also increases. Figure 5b illustrates that although the non-bond energy of the model increases with temperature, the difference in the non-bond energy at different temperatures is small. Therefore, the influence of the thermal field in the temperature range that was used in this work on the van der Waals interactions inside the crystalline regions is small.

**Figure 6:**
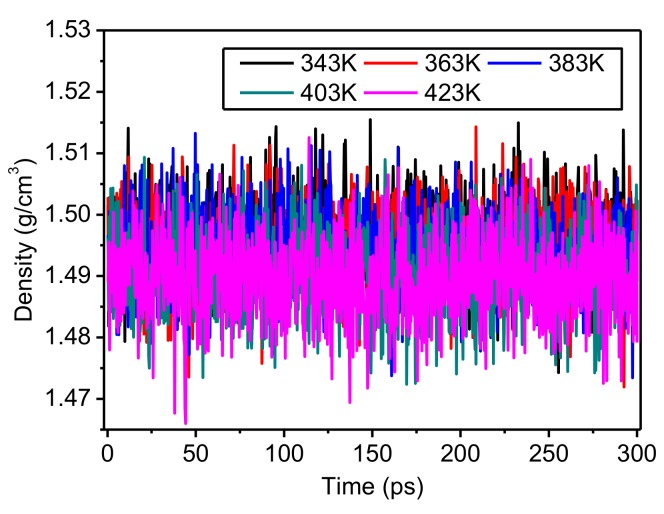
Change of the density in the crystalline regions of meta-aramid fibers during the simulations at different temperatures.

The relationships between the density of the crystalline regions and simulation time at different temperatures are given in Figure 6. Within the simulation time of 300 ps, the density changed within the range of 1.48–1.505 g/cm^3^ without obvious fluctuation, showing again that the model is reliable during this period. The curves overlap because of the small differences in density, indicating that density is not strongly influenced by temperature; in addition, this result also shows that the crystalline regions of meta-aramid fibers have high thermal stability.

Overall, the simulation results showed that the molecular chain motion of crystalline regions of the meta-aramid fibers increased only slightly under the action of a thermal field. The kinetic energy of molecular chains increased with temperature, and the total energy also increased; however, the non-bond energy in the force field expression was not strongly influenced by temperature. The density of crystalline regions remained similar at different temperatures. Therefore, the crystalline regions maintained high thermal stability in the temperature range that was investigated in this paper.

### 4.2. Chain Motion of Amorphous Regions of Meta-Aramid Fibers

**Figure 7:**
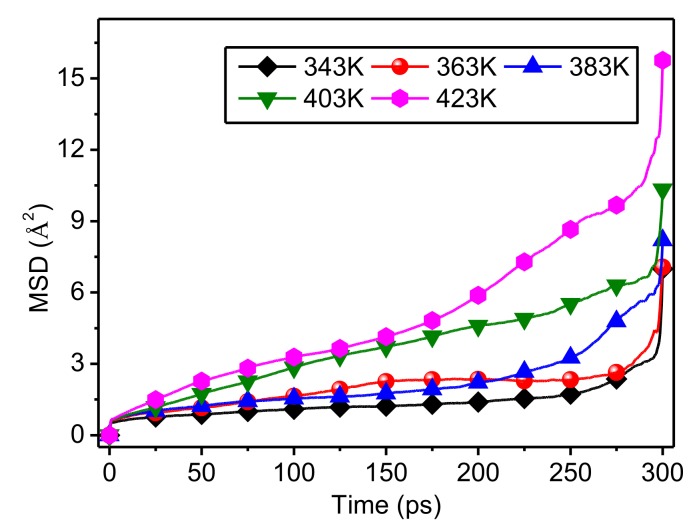
MSD time curves for amorphous regions of meta-aramid fibers at different temperatures.

The MSD time curves for the amorphous regions of meta-aramid fibers at different temperatures are shown in Figure 7. The molecular chain motion of amorphous regions showed a positive correlation with temperature. When the temperature was low, the increase of the MSD of the amorphous regions was slow. Conversely, when the temperature was high, the increase of MSD was rapid, which was manifested as a steeper curve, indicating the stronger molecular chain motion of the amorphous regions at higher temperature. Comparing the curves revealed that the MSD time curves at 343 K and 363 K were similar. After about 225 ps, the higher the temperature, the lower the curve, and the larger the difference between the MSD values of all of the curves. These results show that the molecular chain motion in the amorphous regions is highly sensitive to temperature. Therefore, decreasing the operating temperature of a transformer can effectively improve the thermal stability of amorphous regions of meta-aramid insulation paper.

Comparing the results in Figure 4 and Figure 7 reveals that the chain motions in both the amorphous and crystalline regions of meta-aramid fibers is influenced by temperature; in addition, molecular chain motion and temperature are positively correlated. The MSD values of amorphous regions are obviously higher than those of crystalline regions, showing that the chain motion of amorphous regions in a thermal field is obviously stronger than that of crystalline regions. The sensitivity of the molecular chain motion of amorphous regions to temperature is obviously greater than that of the molecular chains in crystalline regions.

**Figure 8:**
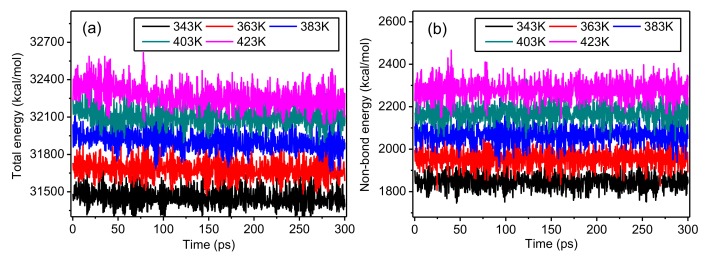
Changes of (**a**) total energy and (**b**) non-bond energy during the simulations at different temperatures.

The changes of total energy and non-bond energy for the amorphous regions of the meta-aramid fibers during the simulations at different temperatures are displayed in Figure 8. With the lengthening simulation time, the model energy remains in dynamic balance; namely, it fluctuates around one stable value, showing that the model conformation was sufficiently stable during the early stage of the simulations. The model energies changed with temperature; the total energy and non-bond energy of the model both increased with temperature, indicating that the temperature strongly influences the model energy during the simulation process. Raising the temperature increased both the aramid chain motion and molecular kinetic energy; in addition, the chain space increased, so the molecular potential energy and total energy also increased, conforming to the laws of thermodynamics. Moreover, the non-bond energy also increased with rising temperature, because the van der Waals force between chains increased with the molecular chain motion.

**Figure 9:**
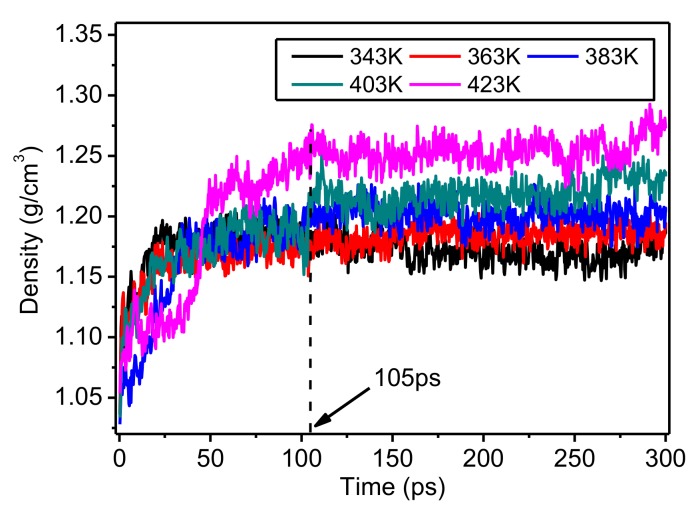
Change of density of amorphous regions of meta-aramid fibers during simulations at different temperatures.

The changes of density of the amorphous regions of meta-aramid fibers during simulations at different temperatures are presented in Figure 9. In the first 105 ps of the simulations, the density of the model increased greatly. After 105 ps, the density did not obviously increase, indicating that the steady-state density is the actual density of the model at a corresponding temperature. The density of the model increased with temperature, which is mainly because the motion of molecular chains strengthened with rising temperature; the model volume also increased. In the NPT ensemble, it is necessary to decrease the model volume V to maintain constant pressure P of particle population N in the model; namely, to fix the model quality. Therefore, the model density was slightly increased. The stronger the motion of molecular chains in the model, the quicker its volume decreases, and the greater the increase of model density. The densities of the model at 343 K, 363 K, and 383 K were similar. In contrast, the densities at 403 K and 423 K were quite different, especially after about 250 ps. This result shows that a higher temperature induces a stronger chain motion in the amorphous regions of the meta-aramid fibers, lowering their thermal stability. Therefore, it is important to minimize the time that a transformer operates at high temperature to maintain the long-term effective operation of meta-aramid insulation paper.

Analyzing the energy changes of the crystalline and amorphous regions of meta-aramid fibers revealed that the molecular kinetic energy increased with the temperature, which caused the total energy to increase with temperature. Considering the microscale energy of a force field, the non-bond energy of crystalline regions is not greatly influenced by temperature, although a strong positive correlation exists between the non-bond energy of crystalline regions and temperature. Further analysis suggested that because the stability of the molecular structure of crystalline regions is high, atomic van der Waals forces in crystalline regions are not greatly influenced by temperature. In contrast, the molecular arrangement of amorphous regions is irregular, and the atom space is easily changed because of the strong motion of molecular chains in a thermal field. As a result, the van der Waals forces are influenced, and the non-bond energy changes. Therefore, the temperature has a stronger influence on the non-bond energy of amorphous regions than that of crystalline regions; similarly, the density change of crystalline regions with temperature is smaller than that of amorphous regions.

The thermal stability of the amorphous regions is obviously poorer than that of the crystalline regions of meta-aramid fibers, which is related to the different molecular structures in these regions. Our results indicated that the aging of meta-aramid insulation paper starts from amorphous regions with relatively poor thermal stability, and the speed of aging is influenced by the thermal field. Increasing the proportion of crystalline regions is an important approach to improve the thermal stability of meta-aramid insulation paper.

## 5. Analysis of Hydrogen Bonding

Hydrogen bonding is a weak force existing between or within molecules that is similar to electrostatic interactions. A hydrogen bond formed between two or more molecules is called an intermolecular hydrogen bond; a hydrogen bond formed between atoms in the same molecule is called an intramolecular hydrogen bond. The hydrogen-bonding network in a polymer influences its melting point, boiling point, structure, and cohesive energy density [43]. It is thought that the mechanical performance and aging resistance of cellulose and other materials are greatly influenced by hydrogen bonding [44]. Intramolecular hydrogen bonding plays vital roles in organisms, such as determining the helical structure of polymers and activity of biochemical molecules [45]. To prepare polymers with high molecular weight, it is necessary to mix various polymers and change the blend composition to regulate performance.

However, polymer blends are difficult to form; instead, most polymers are immiscible and form obvious interfaces with weak intercomponent bonding. Research has shown that the compatibility of immiscible copolymers can be enhanced by forming intermolecular hydrogen bonds, which can improve the performance of polymer blends [46,47]. Researchers have started to focus on the importance of hydrogen bonds since they were first recorded by an experimental method [48]. The definition of a hydrogen bond in this paper agrees with that in the literature [49].

### 5.1. Hydrogen Bonding in Crystalline Regions of Meta-Aramid Fibers

**Figure 10:**
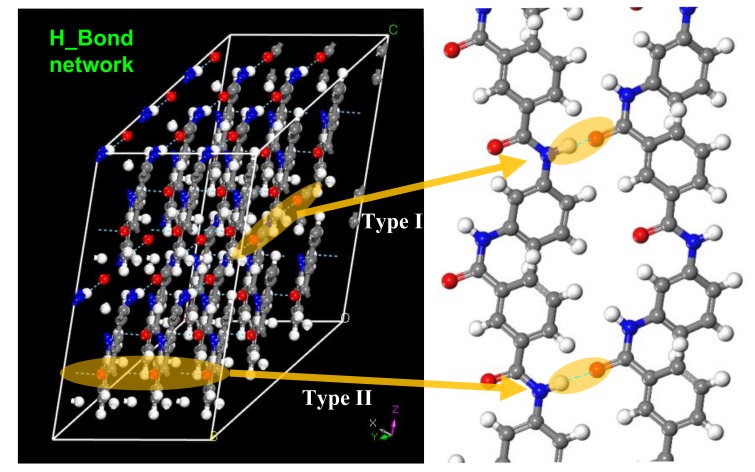
Hydrogen bonding network in the crystalline regions of meta-aramid fibers.

The hydrogen bonds formed in a crystalline area of a meta-aramid fiber are shown in Figure 10. In the crystalline area, only a hydrogen atom bonded to a nitrogen atom forms a hydrogen bond with an oxygen atom on an adjacent chain, giving a hydrogen bond with the form N–H···O, which is the same as that reported elsewhere for a meta-aramid fiber [29]. There are two forms of hydrogen bond in a crystalline area of a meta-aramid fiber [25]: one type is approximately parallel to the a-axis (Type I), and the other type is approximately parallel to the b-axis (Type II). These hydrogen bonds induce the formation of a structure similar to a three-dimensional ladder shape. This type of hydrogen bonding network has only been found in the crystalline regions of meta-aramid fibers. The existence of hydrogen bonds increases the stability of the crystalline regions. The length of Type-I hydrogen bonds is 2.25 Å, and that of Type-II hydrogen bonds is 2.10 Å in the model constructed in this paper.

**Figure 11:**
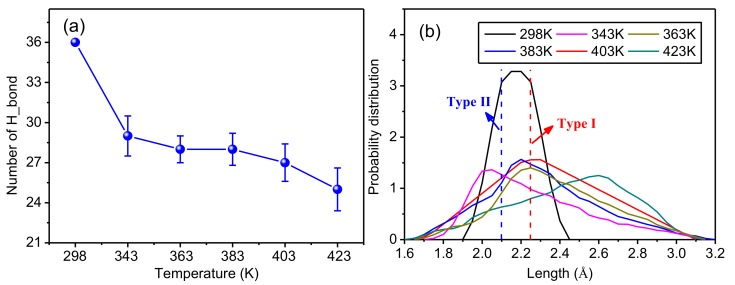
Influence of temperature on the hydrogen bonding network in crystalline regions of meta-aramid fibers. (**a**) Relation between temperature and the number of hydrogen bonds and (**b**) distributions of hydrogen bond lengths at different temperatures.

To further study the influence of temperature on the hydrogen bonding in the crystalline regions of meta-aramid fibers, statistical analysis of the hydrogen bond number and bond distance distribution was conducted; the results are presented in Figure 11a,b. Figure 11a displays the statistical results for five repeated simulations using the initial conformation at 298 K and a fixed number of hydrogen bonds; therefore, there is no error distribution. Figure 11b shows the simulation results closest to the trend of the curve in Figure 11a.

Figure 11a reveals that the number of hydrogen bonds in a crystalline area obviously decreases with rising temperature. This is because the structure of the crystalline area changes with rising temperature; that is, the original structure is unstable. The shift of molecular chains causes the hydrogen bond length to increase. The larger the shift of the molecular chain, the more the bond distance increased until some did not meet the conditions to form a hydrogen bond; therefore, the number of hydrogen bonds decreased with rising temperature. The original hydrogen bonding network inside the crystalline area is thus damaged because of the rising temperature. Since the existence of hydrogen bonds is an important factor maintaining the stability of the structure of crystalline regions of meta-aramid fibers, it is necessary to avoid transformer operation at high temperature for long periods.

Figure 11b shows probability distributions for the hydrogen bond lengths in the model at different temperatures; the larger the ordinate, the larger the probability that a hydrogen bond number occurs with a certain bond length. In the initial configuration, the length of Type-I and Type-II hydrogen bonds in the crystalline regions of meta-aramid fibers is distributed within the range of 1.9 Å to 2.45 Å, conforming to the distribution of actual lengths of these two types of hydrogen bond; because the bond length has only two fixed values, its distribution is the largest. After the dynamics simulations, the hydrogen bond length gradually increases on both sides of the probability distributions; in addition, the number of hydrogen bonds on the right side of the Type-I hydrogen bond is obviously greater than the number of hydrogen bonds on the left side of the Type-II hydrogen bond. That is, the number of long hydrogen bonds is greater than the number of short ones, showing that the original crystal structure has changed to some extent, and the molecular chains have shifted inside the crystalline area. However, a major proportion of the hydrogen bond lengths remained near the Type-I hydrogen bond and Type-II hydrogen bond, showing that the crystal structure has reasonable stability.

Overall, although the structure of the crystalline regions of meta-aramid fibers had pretty high stability, a thermal field may exert a certain influence; in addition, long-term operation at high temperature may degrade the structure of the crystalline regions and influence the stability of the structure of the crystalline regions to unfavorably influence the performance of meta-aramid insulation paper. Therefore, avoiding the operation of transformers at high temperature for long periods is an effective measure to guarantee the performance of meta-aramid insulation paper.

### 5.2. Hydrogen Bonding in Amorphous Regions of Meta-Aramid Fibers

**Figure 12:**
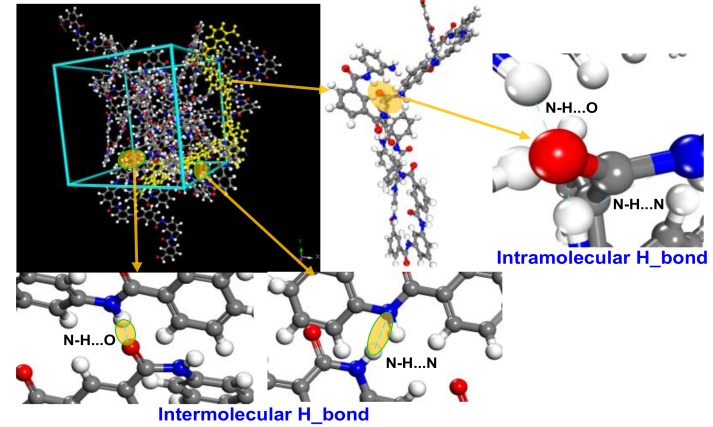
Hydrogen bonding network formed in an amorphous area of a meta-aramid fiber.

The hydrogen bonding network that formed in an amorphous area of a meta-aramid fiber is shown in Figure 12. The hydrogen bond networks formed in the amorphous and crystalline regions are different. In the crystalline area, only the H atom on an –NH radical group and O atom on an adjacent chain formed a hydrogen bond. In addition, the molecular chains in the crystalline regions are well ordered without easy dislocation, distortion, and deformation; therefore, only an intermolecular N-H...O-type hydrogen bond could form. In contrast, the arrangement of molecular chains inside the amorphous regions is irregular, and the long chains are easily deformed, bent, and even folded; therefore, both intermolecular and intramolecular hydrogen bonds can form in the amorphous regions. The number of intermolecular hydrogen bonds was greater than that of intramolecular hydrogen bonds [50]. An N–H···O-type hydrogen bond can form when an O atom is close to an –NH radical group, and an N–H···N-type hydrogen bond can form when an N atom is close enough to an –NH radical group; these two types of hydrogen bond can be intermolecular or intramolecular. In addition, in an amorphous area, many circumstances may occur: one O atom may be close to many –NH radical groups, one –NH radical group may be close to many O atoms, one –NH radical group may be close to many –NH radical groups, or one –NH radical group may be close to an O atom, as well as an –NH radical group. The hydrogen bonds formed through such complex interactions are called unconventional or bifurcated hydrogen bonds [51]. This situation was allowed to exist in this paper.

**Figure 13:**
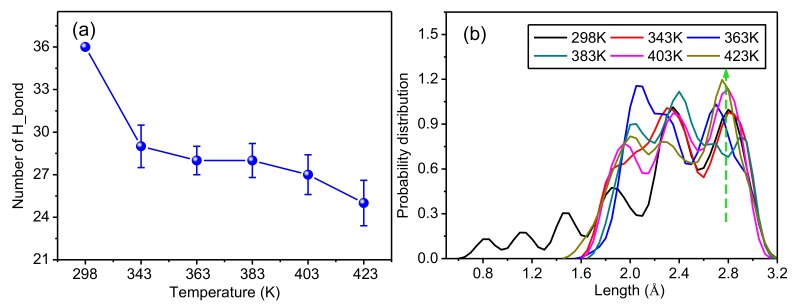
Influence of temperature on the hydrogen bonding network in amorphous regions of meta-aramid fibers. (**a**) Relation between temperature and hydrogen bond number and (**b**) probability distributions of hydrogen bond lengths at different temperatures.

To further analyze the influence of temperature on hydrogen bonding in the model of the amorphous regions of meta-aramid fibers, the relation between hydrogen bond number and temperature is displayed in Figure 13a, which was obtained from repeated simulations. The hydrogen bond number of the model increases with temperature, which is mainly because the motion of fiber molecular chains is enhanced with rising temperature and the intramolecular atom space is changed, which increases the opportunity for intramolecular hydrogen bonds. With the further rise of temperature, the hydrogen bond number decreases slightly, which is mainly because the further rise of temperature further increases the chain space until the original hydrogen bonding network is damaged. In addition, the intramolecular distance can no longer meet the conditions for the formation of a hydrogen bond; therefore, the hydrogen bond number decreases slightly. The hydrogen bond number is similar for the different simulations using the model for the amorphous regions, which is consistent with reported results [50].

Figure 13b shows that the distribution of hydrogen bond lengths at room temperature (298 K) is 0.6 Å to 3.2 Å; compared with the distribution of hydrogen bond lengths at other temperatures, the distribution at room temperature is wider, and there are more short hydrogen bonds. With rising temperature, the number of long hydrogen bonds increases; all curves have pretty high peak values when the bond length is about 2.8 Å. These results also verify that the aramid molecular chain space increases with temperature, resulting in increased atom space, less hydrogen bonds, and increased hydrogen bond length.

## 6. Conclusions

The influence of the thermal field during the operation of a transformer on the thermal stability of meta-aramid insulation paper was studied by a molecular dynamics simulation method. The changes of chemical parameters, chain motion, hydrogen bonding, and other model parameters in crystalline and amorphous regions with temperature were analyzed, which provided the following conclusions:

1. At various temperatures, the modulus values of crystalline regions were two to three times higher than those of amorphous regions. The increased crystallinity of crystalline regions improved the modulus of meta-aramid insulation paper. Toughness may also be influenced to a certain degree. The incompressibility, rigidity, capacity to resist deformation, plasticity, and toughness of the crystalline regions were obviously higher than those of the amorphous areas, whereas the toughness of the amorphous regions was better than that of the crystalline regions. Under the action of a thermal field, the mechanical parameters in the crystalline and amorphous regions of meta-aramid fibers are different. The amorphous regions were more sensitive to temperature than the crystalline regions. The main reason for this difference is molecular structure: the molecular arrangement of crystalline regions had high regularity, whereas the molecular arrangement of amorphous regions had low regularity, and was even random.
2. The action of the thermal field slightly increased the motion of the molecular chains in the crystalline regions of meta-aramid fibers. The molecular chain motion of the amorphous regions was more sensitive to temperature than that in the crystalline regions. The aging of meta-aramid insulation paper starts from amorphous regions with relatively poor thermal stability, and its aging speed is influenced by the thermal field. An appropriate increase of the crystalline area proportion and decrease of the operating temperature of the transformer can improve the thermal stability of meta-aramid insulation paper.
3. Long-term operation at high temperature may destroy the structure of crystalline regions of meta-aramid fibers, lowering their stability and degrading the performance of meta-aramid insulation paper. The rise of temperature may change the distributions of hydrogen bond lengths in both the crystalline and amorphous regions. Type-I and Type-II hydrogen bonds were observed in the crystalline regions. The hydrogen bond length in amorphous regions increased and then gradually decreased with rising temperature.

## Figures and Tables

**Table 1:** Mechanical parameters (GPa) of the crystalline regions of meta-aramid fibers at different temperatures.

	343 K	363 K	383 K	403 K	423 K
C_11_	16.0766	15.6049	15.8853	15.4653	15.9172
C_22_	23.9779	22.4932	23.3769	21.8053	23.8483
C_33_	125.3986	123.4178	124.6375	122.6217	125.2992
C_44_	4.5859	4.3149	4.3826	4.2938	4.4550
C_55_	10.8213	10.2667	10.7308	9.9972	10.7810
C_66_	4.9536	4.5564	4.8864	4.5042	4.9309
C_12_	13.0176	12.3408	12.7655	12.0673	12.9137
C_13_	11.8826	10.9720	11.5453	10.2754	11.7574
C_23_	11.0271	9.8351	10.5568	9.2691	10.9485
C_15_	0.7719	0.7950	0.7519	0.9097	0.7905
C_25_	2.2807	2.1310	2.2902	2.1407	2.2932
C_35_	-12.6427	-11.4554	-12.1603	-10.8019	-12.6015
C_46_	-1.2672	-1.2086	-1.2434	-1.1815	-1.2688
*K*	15.0370	14.4768	14.8211	14.2173	14.8985
*G*	4.5149	4.3324	4.4061	4.2939	4.4462
*E*	12.3125	11.8183	12.0265	11.7034	12.1319
*ν*	0.3635	0.3639	0.3648	0.3628	0.3643
C_12_–C_44_	0.2380	0.2498	0.2445	0.2516	0.2424
*K/G*	3.3305	3.3415	4.4061	3.3111	3.3508

**Table 2:** Mechanical parameters (GPa) of the amorphous regions of meta-aramid fibers at different temperatures.

	343 K	363 K	383 K	403 K	423 K
C_11_	8.1447	7.9929	5.1929	5.6865	6.8282
C_22_	8.9980	7.5829	8.4336	7.0976	6.3522
C_33_	7.2241	5.9593	4.9543	4.5814	2.0924
C_44_	2.2998	1.7513	1.8466	2.2429	1.5376
C_55_	2.0434	1.2013	1.6225	0.9254	0.9510
C_66_	2.8573	2.0955	1.7349	1.6217	1.8102
C_12_	3.8344	2.9190	3.0527	2.4052	2.5152
C_13_	2.4708	2.1749	1.7479	1.6032	0.8586
C_23_	3.6596	2.4105	2.5815	2.3323	1.4437
C_15_	-0.0694	0.8525	-0.0942	0.3096	0.5644
C_25_	-0.3623	0.5968	-0.0516	-0.3104	0.0129
C_35_	0.1970	0.9408	-0.1131	-0.3859	0.4334
C_46_	-0.0433	0.0407	0.0788	-0.0223	-0.1534
*K*	4.6983	3.3653	3.2997	3.1083	1.5984
*G*	2.3164	1.7035	1.7287	1.3537	1.1772
*E*	5.9683	4.3728	4.4152	3.5463	2.8354
*ν*	0.2883	0.2834	0.2770	0.3098	0.2043
C_12_–C_44_	0.4320	0.6818	0.5838	0.8279	0.8389
*K/G*	2.0283	1.9755	1.9087	2.2961	1.3578
